# Evaluation of adding the Erector spinae plane block to standard anesthetic care in patients undergoing posterior lumbar interbody fusion surgery

**DOI:** 10.1038/s41598-021-87374-w

**Published:** 2021-04-07

**Authors:** Renee J. C. van den Broek, Robbin van de Geer, Niek C. Schepel, Wai-Yan Liu, R. Arthur Bouwman, Barbara Versyck

**Affiliations:** 1 Department of Anesthesiology, Intensive Care and Pain Medicine, Catharina Hospital, Michelangelolaan 2, 5623 EJ Eindhoven, The Netherlands; 2 Department of Orthopedic Surgery and Trauma, Catharina Hospital, Michelangelolaan 2, 5623 EJ Eindhoven, The Netherlands; 3 Department of Anesthesiology, AZ Turnhout, Campus St Jozef, Steenweg op Merksplas 44, 2300 Turnhout, Belgium

**Keywords:** Physiology, Medical research

## Abstract

Postoperative analgesia in patients undergoing spinal fusion surgery is challenging due to the invasiveness of the surgical procedure and the frequent use of opioids preoperatively by many patients. Recently, the erector spinae plane (ESP) block has been introduced in our clinical practice as part of a multimodal pain strategy after posterior lumbar interbody fusion surgery. This is a retrospective case–control study evaluating the analgesic efficacy of the ESP block when added to our standard analgesic regimen for posterior lumbar interbody fusion surgery. Twenty patients who received an erector spinae plane block were compared with 20 controls. The primary endpoint was postoperative pain, measured by the numeric rating scale. Secondary outcome measures were opioid use, postoperative nausea and vomiting, and length of stay. Postoperative pain scores in the PACU were lower in patients who received an erector spinae plane block (*p* = 0.041). Opioid consumption during surgery and in the PACU was not significantly different. Need for patient-controlled analgesia postoperatively was significantly lower in the group receiving an ESP block (*p* = 0.010). Length of stay in hospital was reduced from 3.23 days (IQR 1.1) in the control group to 2.74 days (IQR 1.6) in the study group (*p* = 0.012). Adding an erector spinae plane block to the analgesic regimen for posterior lumbar interbody fusion surgery seemed to reduce postoperative pain and length of hospital stay.

## Introduction

Patients can experience severe postoperative pain after spine surgery. A study comparing pain intensity on the first postoperative day in 179 different surgical procedures ranked spinal fusion surgery at second place, after open reduction of calcaneus fracture^1^. Postoperative pain management in spinal fusion surgery is challenging and usually includes administration of extensive amounts of opioids^2^. This can cause well known side-effects, such as respiratory depression, sedation, nausea, vomiting, and constipation. These side-effects can lead to a longer hospital stay and a worse patient experience^3,4^. Even with opioids, pain is not always sufficiently managed. Inadequate pain control increases cardiac and respiratory complications, delays mobilization, increases the length of hospital stay and may increase the risk of developing a chronic pain syndrome^5^.

The above mentioned complications indicate the need for a multimodal analgesic approach to posterior lumbar interbody fusion (PLIF) surgery with an increasing role for novel regional anesthesia techniques. Until recently, regional anesthesia techniques have not been used on a regular basis in PLIF surgery as an immediate postoperative neurological examination is required. This examination of the motor and sensory function of the spinal cord eliminates spinal and epidural analgesia as suitable pain treatments. Novel interfascial plane blocks, such as the erector spinae plane (ESP) block, generate regional analgesia without interference of spinal cord function and are therefore suitable for spinal surgery pain management.

The ESP block was first described in 2016^6^. Using ultrasound, local anesthetic is injected below the erector spinae muscle group (m. spinalis, m. longissimus thoracis and m. ileocostalis)^6,7^. This causes a sensory blockade over the antero- and dorsolateral thorax by blocking ventral and dorsal rami of the spinal nerves. Recent case reports suggest a positive effect of an ESP block on pain for multiple indications including vertebral metastases, lumbar transverse process fractures or following lumbar spine fusion and scoliosis surgery^8–11^. An ESP block has a very low risk of complications, as sonoanatomy is easily recognizable and there are no structures in close proximity at risk of needle injury^6,12^. The transverse process acts as an anatomical barrier and avoids needle insertion into the pleura or vessels, thus preventing a pneumothorax or hematoma. Moreover, the needle is relatively far from the vertebral canal, which means the risk of spinal cord injury is very low. In a pooled review, which yielded 242 reported cases between 2016 and 2018, only one adverse event (a pneumothorax) was reported^13^. An ESP block preserves bladder function and motor neuron function enabling early mobilization. Since motor function is unaltered, immediate postoperative neurological evaluation of spinal cord function is possible.

We hypothesized that for patients undergoing PLIF surgery, adding an ESP block to standard anesthesia treatment might provide additional analgesia and reduce the need for postoperative opioids. We implemented this block in our clinical practice in 2018. This study aimed to retrospectively evaluate pain scores during the first 24 h post-surgery. We examined the effect of the ESP block on opioid consumption during the first 24 h post-surgery, the occurrence of postoperative nausea and vomiting (PONV), the need for patient-controlled analgesia (PCA) and length of stay (LOS) as secondary outcome parameters. Complications registered as part of standard registration of care were evaluated as well.

## Materials and methods

This is an observational retrospective case–control study. It was approved by the Medical Research Ethics Committees United (MEC-U, St. Antonius Hospital, Koekoekslaan 1, PO Box 2500, 3430 EM Nieuwegein, the Netherlands) (document number W20.100). The study was performed in accordance with the Declaration of Helsinki (Fortaleza, Brazil, October 2013) and in accordance with the Medical Research Involving Human Subjects Act (WMO) and Good Clinical Practice guidelines. We followed the STROBE (STrengthening the Reporting of OBservational studies in Epidemiology) guideline to report this study.

### Case and control selection

From September 2019 until the end of January 2020, we performed the ESP block in 20 patients undergoing PLIF surgery. These blocks were performed by anesthesiologists with clinical expertise in regional anesthesia. During the preoperative consultation, informed consent was obtained for supplementary analgesia by ESP block. The study patients were the last 20 patients who underwent PLIF surgery at the moment we decided to undertake this evaluation. Twenty controls were selected from an earlier period (June 2017–January 2018), when use of the ESP block or any other local anesthetic was not part of standard care for this procedure. All patients in this study were treated by the same two surgeons. Consent for the anonymous use of the data of all patients was obtained by a letter asking for permission, according to national general data protection regulation and in consultation with the hospitals’ privacy officer.

### Surgery

The goal of PLIF surgery is to decompress painful nerve roots and permanently immobilize adjoining vertebrae of the lumbar spine. The indications for PLIF surgery were persistent radicular pain due to either spondylolisthesis, lateral foraminal stenosis, failure of previous neurosurgical decompression, or a combination of the above. All patients underwent conservative treatment before surgery i.e. physiotherapy, oral analgesics, sleeve infiltration and/or pulsed radiofrequency therapy.

All operations performed by a surgical team comprising of an orthopedic and a neurosurgeon. The PLIF procedure was first described in 1944 and has evolved to the current method as described in a review by Mobbs^14^. Access was obtained in supine position on Wilson frame through a midline incision followed by subperiosteal dissection of the spinal musculature until the transverse processes of corresponding vertebrae were reached. Titanium pedicle screws (Expedium Depuy-Synthes, Raynham, United States) where placed before laminectomy and decompression of affected nerves was performed. After discectomy, PLIF cages (Plivios Depuy-Synthes, Raynham, United States) and autologous bone were introduced between the denuded endplates of the disc space. Nerve decompression and introduction of PLIF cages was always performed in cooperation with a neurosurgeon. Pedicle screws were fixed to titanium rods thus obtaining a rigid 360 degree fixation. Fascia, subcutis and skin were closed separately with dissolvable stitches. There was a no drain policy.

Patients were permitted to walk 4 h postoperatively. They were discharged home when able to walk the stairs and if pain was manageable with oral analgesics.

### Perioperative management

Preoperatively, all patients were given an intravenous (IV) canula and received oral administration of paracetamol if contra-indications were absent. Standard monitoring as advised by the American Society of Anesthesiologists (ASA) was applied. After completing the safety procedures, general anesthesia was induced. Patients were placed in prone position for surgery. Patients received sufentanil at induction and during surgery. Some patients received a bolus of morphine at the end of surgery, depending on the preference of the attending anesthesiologist.

After surgery, patients were admitted to the post anesthesia care unit (PACU). In the PACU, patients were clinically assessed routinely for postoperative complications. Numeric rating scale (NRS) scores for pain and presence of PONV were documented by the PACU nurses. In case of a NRS pain score of more than 3, the patient received intravenous boluses of morphine until pain relief (NRS < 4) was achieved. This was performed according to the standard hospital’s postoperative pain protocol.

Patients were discharged from the PACU when Aldrete’s scores were 8 or above, NRS pain scores 3 or lower, and PONV was absent or adequately treated. On the ward, a medical assessment including NRS pain scores was documented by the nurses at set intervals. All postoperatively administered medication was documented in the clinical file.

### Erector spinae plane block

The ESP block was performed by administering local anesthetic beneath the erector spinae muscle group. In a lumbar ESP block, local anaesthetics may spread to the lumbar plexus resulting in motor weakness as demonstrated in radiologic studies^15–17^. Therefore, we chose to perform the ESP block at the lowest thoracic level.

The ESP block was performed after induction of anesthesia and after placement of the patient in prone position for surgery. The ESP block was performed as described by Forero et al.^6^. By using ultrasound (Philips Sparq, Amsterdam, the Netherlands) with a high-frequency curved array probe (Philips IPx-7 C5-1 PureWave, Amsterdam, The Netherlands), the erector spinae muscles were identified in relation to the transverse processes of T12. The probe was placed 2–3 cm lateral to the vertebral column, in longitudinal alignment. A 10-cm 21 gauge ultrasound-needle (Pajunk SonoPlex STIM, Geisingen, Germany) was inserted in-plane in a cephalad to caudal direction. After bone contact with the transverse process was obtained, the needle was retracted slightly. Hydrodissection with normal saline (NaCl 0.9%) was performed to identify and open up the correct plane. After confirmation of correct placement of the needle, a dose of 20 mL of ropivacaine hydrochloride was injected. The same procedure was performed on the contralateral side.

Patients over 70 kg received 200 mg ropivacaine (40 ml), patients 50–70 kg received 150 mg ropivacaine (40 ml), and patients under 50 kg received ropivacaine 3 mg/kg (40 ml).

### Data collection

All data were collected from the hospital’s patient data management systems. Preoperative data were collected from the preoperative anesthesia screening and the surgical consultations. During surgery, registration of medication and procedures regarding analgesia and anesthesia was performed by the attending anesthesiologist. Nurses on the PACU and surgical ward documented the patient’s condition as part of standard practice. We collected data from 4 different time points postoperatively: at the PACU and after 6, 12 and 24 h postoperatively. We compared both groups by calculating the oral morphine equivalent (OME) of all opioids used during surgery^18^. In the PACU, only morphine was administered and therefore not converted to OME.

### Statistical analysis

Our primary outcome was postoperative NRS pain scores. Secondary outcome parameters were opioid use, PONV, and LOS. Continuous variables were presented as mean and standard deviation (SD) or median and interquartile range (IQR), depending on normality. Categorical demographic variables were reported as a number or a percentage.

Differences in normal distributed continuous variables between groups were tested using an independent T-test. Differences in not-normal distributed continuous variables between groups were tested using a Mann–Whitney U-test. Differences in categorical variables between groups were tested using a Fisher’s exact test. A *p*-value < 0.05 was considered statistically significant when comparing the two groups. We have calculated the effect size for nonparametric data using r = z/√N, as described by Fritz, Morris and Richler (2012). The effect size was calculated such as the r proposed by Cohen (1988); 0.5 for a large effect; 0.3 for a medium effect and 0.1 for a small effect. Statistical analyses were performed using SPSS, version 25 (SPSS Inc., Chicago IL).

### Consent to participate

Informed consent was obtained from all individual participants included in the study.

## Results

The demographic data including age, sex, ASA classification, BMI, history of spine surgery, number of levels, and length of surgery, were equally distributed between the two groups (Table 1).

**Table 1:** Demographics.

	No block (n = 20)	ESP block (n = 20)	*p*-value
Mean age (SD) in years	62.9 (10.9)	61.6 (9.5)	0.680
**Sex, n (%)**			0.341
Male	7 (35)	11 (55)	
Female	13 (65)	9 (45)	
**ASA, n (%)**			0.741
I	1 (5)	1 (5)	
II	14 (70)	11 (55)	
III	5 (25)	8 (40)	
Mean BMI (SD) in kg/m^2^	27.0 (4.0)	26.7 (4.6)	0.830
Mean length of surgery (SD) in min	247 (46)	228 (29)	0.135
**History of spine surgery, n (%)**			0.191
Yes	10 (50)	15 (75)	
No	10 (50)	5 (25)	
**Levels of surgery, n (%)**			1.000
1	14 (70)	14 (70)	
2	6 (30)	6 (30)	

SD, Standard deviation: ASA, American Society of Anesthesiologists. An independent samples T-test was used to compare means; for categorical variables a Fisher’s exact test was used.

Postoperative NRS pain scores in the PACU were lower in patients who received an ESP block (no block: median 5, IQR 6.0; ESP block: median 2, IQR 5.0; *p* = 0.040, with a medium effect; r =  − 0.330). On the ward, NRS pain scores were comparable between the 2 groups after 6 h (no block: median 3, IQR 5.0; ESP block median 2, IQR 2.0; *p* = 0.800, with a small effect; r =  − 0.042), 12 h (no block: median 3, IQR 2.0; ESP block: median 3, IQR 2.0; *p* = 0.458, with a small effect; r =  − 0.121) and 24 h (no block: median 3, IQR 3.0; ESP block 3, IQR 5.0; *p* = 0.444, with a small effect; r =  − 0.132).

Opioid administration during surgery was comparable between the groups (Table 2). In the PACU, patients received only intravenous morphine, no other opioids were administered. There was a trend towards less intravenous morphine administration in the PACU in the ESP block group, but statistical significance was not reached (Table 2).

**Table 2:** Opioid use, recovery time and length of stay.

	No block(n = 20)		ESP block (n = 20)		*p*-value	r
	Median	IQR	Median	IQR		
Oral Morphine Equivalent during surgery (mg)	115	56.3	105	32.5	0.166	− 0.221
Morphine use at PACU (mg)	9	10.5	7	11.9	0.639	− 0.076
Recovery time (min)	67	36.0	67	47.0	0.499	− 0.109
Length of stay (days)	3.23	1.1	2.74	1.6	0.012	− 0.394

IQR, Inter Quartile RangeA Mann–Whitney U-test was used.

LOS was significantly reduced in the ESP block group.

PONV occurred in 2 cases (11.1%) in the control group, compared to 0 cases in the ESP block group (*p* = 0.486, with a small effect; phi coefficient = 0.239). The percentage of patients needing patient-controlled analgesia postoperatively differed between the groups (no block: 75%; ESP block: 30%; *p* = 0.010). Patients who did not need a PCA pump, received intermittent oral opioids (oxycodone). There was no registration of the amount of morphine used by patients who had a PCA pump. Therefore, the collected data did not allow for a meaningful comparison between the PCA and oral analgesic group. There were no ESP block related complications mentioned in the charts. One case of ileus was registered in the ESP block group. This patient needed PCA with intravenous morphine postoperatively and was limited in mobilization due to wound leakage.

## Discussion

This study evaluated the introduction of the ESP block to standard anesthetic care for PLIF surgery. Our results show lower NRS pain scores directly postoperatively in the ESP group. With comparable administration of opioids in both groups during surgery and in the PACU, adding an ESP block to the analgesic regimen for PLIF surgery seemed to reduce pain in the PACU. Our results were consistent with two randomized trials that compared the ESP block to standard treatment in patients undergoing PLIF surgery and showed a positive effect on pain and a reduction of opioid consumption^19,20^. Although we did not measure postoperative opioid consumption by PCA pump on the ward, the reduction in need for PCA with intravenous morphine suggests less need for pain management with opioids. Less intravenous opioids will contribute to earlier postoperative mobilization and has a positive effect on recovery^21^. We therefore hypothesize that this mechanism is the reason for the reduction in LOS in our study.

Multimodal pain regimens, such as described by Dietz et al. in their guidelines of enhanced recovery after surgery in spine surgery, are based on different pathways to reduce pain^22^. The addition of an ESP block to general anesthesia fits perfectly within this paradigm. In the ESP block, local anesthetic spreads within the musculofascial plane deep to the erector spinae muscle and acts on the dorsal rami of spinal nerves at multiple levels. The branches of the dorsal rami innervate the paraspinal muscles and the vertebrae itself. Evidence indicates that 20 ml of injectate extends 3–8 vertebral levels^23^. An ESP block therefore diversifies the pain management with an additional pathway.

Length of stay for lumbar spine fusion surgery has been reduced dramatically by enhanced recovery after surgery (ERAS)-protocols in the last decade. A retrospective study found a reduction from 6.7 days in 2012–2013 to 4.8 days in 2016–2017 for posterior lumbar spine fusion^24^. In our center, median LOS for the control group was 3.2 days. Hospital stay was significantly reduced after implementation of the ESP block to 2.7 days. Furthermore, compliance to early mobilization protocols is hampered by uncontrolled pain^25^. By optimizing our pain protocol we have significantly reduced the LOS. In addition to the improvement of patient care, this also results in reduced hospital costs and higher hospital bed capacity^26,27^.

Our data show that the time spent in the operating theatre did not differ between the groups, as the ESP block is easily performed while the surgeon is scrubbing in for surgery. As the ESP block was performed after general anesthesia was induced, there was no patient discomfort during performance of the block. Implementation of this block as standard care was easy in our center and we did not find disadvantages for our patients.

This study has two limitations. Firstly, this is a small-scale retrospective clinical evaluation. Hence, sample size was not a priori powered and our conclusion cannot be generalized. Secondly, staff followed our in-house protocol which allowed a more liberal anesthesia regimen and resulted in less detailed data registration compared with a strict study protocol.

Implementing the ESP block for PLIF surgery as standard care in our center has caused a significant reduction in postoperative pain and length of hospital stay, most likely by allowing earlier mobilization. As we have increased our experience with the ESP block, these results encourage us to perform a large prospective, randomized clinical trial to further establish the role of the ESP block in PLIF surgery.

## Supplementary Information

Supplementary Information

## Data Availability

The dataset generated and analyzed during the current study is available; it is added to the submission of this manuscript as a separate file.

## Abbreviations

ASA: American Society of Anesthesiologists
BMI: Body mass index
ERAS: Enhanced recovery after surgery
ESP: Erector spinae plane
IQR: Inter quartile range
IV: Intravenous
LOS: Length of stay
NRS: Numeric rating scale
PACU: Post anaesthesia care unit
PCA: Patient controlled analgesia
PLIF: Posterior lumbar interbody fusion
PONV: Postoperative nausea and vomiting
SD: Standard deviation
WMO: Dutch abbreviation: Medical Research Involving Human Subjects Act

## References

[1] Gerbershagen HJ (2013). Pain intensity on the first day after surgery: a prospective cohort study comparing 179 surgical procedures. Anesthesiology.

[2] Bajwa SJ, Haldar R (2015). Pain management following spinal surgeries: an appraisal of the available options. J. Craniovert. Jun Spine.

[3] Cozowicz C (2017). Opioid prescription levels and postoperative outcomes in orthopedic surgery. Pain.

[4] Soliman IE, Apuya JS, Fertal KM, Simpson PM, Tobias JD (2009). Intravenous versus epidural analgesia after surgical repair of pectus excavatum. Am. J. Ther..

[5] Thepsoparn M, Sereeyotin J, Pannangpetch P (2018). Effects of combined lower thoracic epidural/general anesthesia on pain control in patients undergoing elective lumbar spine surgery: a randomized controlled trial. Spine.

[6] Forero M, Adhikary SD, Lopez H, Tsui C, Chin KJ (2016). The erector spinae plane block: a novel analgesic technique in thoracic neuropathic pain. Reg. Anesth. Pain Med..

[7] Ivanusic J, Konishi Y, Barrington MJ (2018). A cadaveric study investigating the mechanism of action of erector spinae blockade. Reg. Anesth. Pain Med..

[8] Ahiskalioglu A (2018). Erector spinae plane block for bilateral lumbar transverse process fracture in emergency department: a new indication. Am. J. Em. Med..

[9] Altıparmak B, Korkmaz TM, Uysal Aİ, Gümüş DS (2019). Bi-level erector spinae plane block for the control of severe back pain related to vertebral metastasis. BMJ Case Rep..

[10] Almeida CR, Oliveira AR, Cunha P (2019). Continuous bilateral erector of spine plane block at T8 for extensive lumbar spine fusion surgery: case report. Pain Pract..

[11] Chin KJ, Dinsmore MJ, Lewis S, Chan V (2019). Opioid-sparing multimodal analgesia with bilateral bi-level erector spinae plane blocks in scoliosis surgery: a case report of two patients. Eur. Spine J..

[12] Beek J., Smits R.J.H., Fenten M.G.E., Filippini-de Moor G.P.G. ESPB: Het Erector Spinae Plane-Blok. *A&I*. **12**, 50–53 (2019). Accessed April 5, 2019. https://www.a-en-i.nl/tijdschrift/editie/t/editie-1-2019-8

[13] Tsui BCH, Fonseca A, Munshey F, McFadyen G, Caruso TJ (2019). The erector spinae plane (ESP) block: a pooled review of 242 cases. J. Clin. Anesth..

[14] Mobbs RJ, Phan K, Malham G, Seex K, Rao PJ (2015). Lumbar interbody fusion: techniques, indications and comparison of interbody fusion options including PLIF, TLIF, MI-TLIF, OLIF/ATP, LLIF and ALIF. J. Spine Surg..

[15] Tulgar S, Selvi O, Senturk O, Serifsoy TE, Thomas DT (2019). Ultrasound-guided erector spinae plane block: indications, complications, and effects on acute and chronic pain based on a single-center experience. Cureus.

[16] Tulgar S, Senturk O (2018). Ultrasound guided Erector Spinae Plane block at L-4 transverse process level provides effective postoperative analgesia for total hip arthroplasty. J. Clin. Anesth..

[17] Tulgar S (2018). Clinical experiences of ultrasound-guided lumbar erector spinae plane block for hip joint and proximal femur surgeries. J. Clin. Anesth..

[18] Hoban B. *Comparing Opioids: A Guide to Estimating Oral Morphine Equivalents (OME) in Research* (2015). 10.13140/RG.2.1.1297.0729

[19] Singh S, Choudhary NK, Lalin D, Verma VK (2020). Bilateral ultrasound-guided erector spinae plane block for postoperative analgesia in lumbar spine surgery: a randomized control trial. J. Neurosurg. Anesthesiol..

[20] Yayik AM (2019). Postoperative analgesic efficacy of the ultrasound-guided erector spinae plane block in patients undergoing lumbar spinal decompression surgery: a randomized controlled study. World Neurosurgery..

[21] Cheung CW, Wong SSC, Qiu Q, Wang X (2017). Oral oxycodone for acute postoperative pain: a review of clinical trials. Pain Phys..

[22] Dietz N (2019). Enhanced recovery after surgery (ERAS) for spine surgery: a systematic review. World Neurosurg..

[23] De Cassai A, Tonetti T (2018). Local anesthetic spread during erector spinae plane block. J. Clin. Anesth..

[24] Debono B (2019). Benefits of enhanced recovery after surgery for fusion in degenerative spine surgery: impact on outcome, length of stay, and patient satisfaction. Neurosurg. Focus.

[25] Burgess LC, Wainwright TW (2019). What is the evidence for early mobilisation in elective spine surgery? A narrative review. Healthcare.

[26] Shields LBE, Clark L, Glassman S, Shields C (2017). Decreasing hospital length of stay following lumbar fusion utilizing multidisciplinary committee meetings involving surgeons and other caretakers. Surg. Neurol. Int..

[27] Fawcett WJ, Mythen MG, Scott MJPI (2012). Enhanced recovery: more than just reducing length of stay?. Br. J. Anaesth..

